# Everybody's Page

**Published:** 1889-09-28

**Authors:** 


### Everybody's Page.

Some people try to cure, or at east deaden, the friskiness of vicious horses by dosing them with opium.

---

The latest development of the automatic machine is calculated to satisfy the most avaricious. It supplies correct weight, a sweetmeat, and a portrait, all for a penny.

---

The Irish Pasteur, M'Govern, in whose family tradition has handed down a successful cure for hydrophobia, recently received £12 from the Newry Board of Guardians for curing four patients sent to him by them.

---

Bishop Berkeley set all England drinking tar-water in 1744, by his praise of it as a specific for every disease under the sun. It was supposed to be specially beneficial in small-pox, both as a preventive and a cure. It was proper to take half a pint before breakfast and another half pint after supper. The decoction does not sound as if it would be appetising, but some people got to like it, and continued taking it when they were in perfect health.

---

An American dentist surprised his colleagues at the Brighton Congress by stating that he could extract teeth painlessly without making the patient unconscious. His method was to make them breath very rapidly, taking about 200 inspirations a minute. He was allowed to demonstrate the process at the Dental Hospital next day; but apparently the patients did not breathe with the desired rate of rapidity. At least, they did not find the operation painless.

---

Doctors, when removing to a new abode, please don't leave poisonous drugs in the old one. Recently a family moved into a new house, and in looking through the cupboards, the three children of the family found a pot containing what they thought was treacle. Naturally they began eating it, but soon felt ill-effects. They were taken to University College Hospital, where the doctors discovered that the supposed treacle was belladonna, and promptly applied the necessary remedies.

---

Elixirs of life are dangerous things to meddle with. Without going into the realm of fiction and quoting the fate of Mr. Rider Haggard's heroine, an incident recently reported from America may be quoted. A doctor in Kentucky prepared a dose of elixir of life according to the formula of Dr. Brown-Séquard, recently published. He injected it into a decrepit old negro, who presumably wished to be young again. Within a short time the negro died, the symptoms being those of blood poisoning. The doctor fled, and, as nothing has been heard of him, it is supposed that the negro's friends have revenged his death on the doctor.

---

Mrs. Crawford, the charming *doyenne* of lady journalists, quotes the opinion of a gardener at the botanical gardens at Verrières regarding the obstinacy and self-will of plants. "They talk of the difficulty of keeping the canine races pure," he said. "It is child's play compared with that of keeping plants from being crossed with kindred species. They do not want to be too handsome or too pulpy, or succulent, and they are always trying to revert to hardy primitive type. Such types defy the insect world, and are a protection from human beings, not being of much use to them." The plants don't want to be improved against their will, and often, in spite of all precautions, form *mésalliance*. The punishment of this crime is prompt cremation, and the number of criminals thus disposed of every year is enormous.

---

Antipyrin has developed a disease of its own. It has had a poisonous action in some cases, producing what is called "antip rin epilepsy."

---

The German Government propose forming a botanical garden in the Cameroons, for the purpose of determining what plants, medicinal or commercial, are the best for cultivation in the plantations of Germany's African colonies.

---

Fly-papers are just now very plentiful in Missouri. The editor of the *St. Louis County Sentinel* having observed a diminution of public interest in his journal during the hot season, has determined to give it an element of usefulness by printing it on a duly arsenicated fly-paper.

---

At Avala, near Belgrade, quicksilver is abundant in conjunction with a green-coloured mineral named avalite, the properties of which are still unknown. It is hoped that amongst other uses, it may be found possible to apply it as a substitute for arsenic as a colouring body, in which event the discovery will prove very valuable, as avalite is said to be free from the poisonous qualities which make the employment of arsenic so dangerous. The mine was discovered by Professor Klerics, of Belgrade.

---

It is all very well to cry out against dangerous trades, and say they ought to be suppressed. But none of these trades are followed except with the intention of producing some necessary article. Improve the conditions of labour if you will, let machinery come in to supplement hand labour wherever it is possible. But to suppress the industry means to deprive one part of society of an article it requires—be it coal, white lead, scissors, or anything else; and it is doubtful if the other, the workmen themselves, will thank you for ruining their trade. They are more likely to complain, in the words of the illogical patient, that they don't want to be starved to death for the sake of living a little longer.

---

The first ancestor of the bicycle and tricycle, the velocipede, was born in France, in the stormy days of the Revolution, and was popular with the Incroyables, the mashers of the Directorate. Their machine was a very simple and even clumsy one. There were no pedals attached to the wheels, these were not invented till 1855. The rider struck each foot in turn against the ground with force enough to set the two low wheels of the machine rotating, and by this fatiguing mode of progress attained a considerable speed. But he would never have thought of setting himself to race a carriage with any hope of success. What would the Incroyable think of the smooth, swift, silent bicycle of to-day? What could he do but exclaim "Incroyable."

---

A well-known American patent medicine firm have lately been distributing broadcast, from door to door, packages containing a sample of their pills and corn plasters. A correspondent, writing to one of the Liverpool daily papers, complains of this dangerous mode of delivery. He states: "A box of pills containing five, and a corn plaster enclosed in a small packet, was pushed through the letter-box of my door, which got into the hands of one of my children, a little girl of three years, who had innocently taken two of the pills before being detected." He suggests that the persons employed should see the door answered and the packets properly delivered, so that they might not fall into the hands of children who might use them with dangerous results.

